# The emerging concern of IMP variants being resistant to the only IMP-type metallo-β-lactamase inhibitor, xeruborbactam

**DOI:** 10.1128/aac.00297-25

**Published:** 2025-06-09

**Authors:** Christophe Le Terrier, Salvador I. Drusin, Patrice Nordmann, Johann Pitout, Gisele Peirano, Alejandro J. Vila, Diego M. Moreno, Laurent Poirel

**Affiliations:** 1 Medical and Molecular Microbiology, Faculty of Science and Medicine, University of Fribourg 98839, Fribourg, Switzerland; 2 Division of Intensive Care, Department of Acute Care Medicine, Geneva University Hospitals 30578, Geneva, Switzerland; 3 Department of Anaesthesiology, Pharmacology, Intensive Care and Emergency Medicine, Faculty of Medicine, University of Geneva 28535, Geneva, Switzerland; 4 Facultad de Ciencias Bioquímicas y Farmacéuticas, Universidad Nacional de Rosario 63029 , Rosario, Santa Fe, Argentina; 5 Instituto de Química Rosario (IQUIR, CONICET-UNR), Rosario, Santa Fe, Argentina; 6 Swiss National Reference Center for Emerging Antibiotic Resistance, Fribourg, Switzerland; 7 Cummings School of Medicine, University of Calgary 70401, Calgary, Alberta, Canada; 8 Alberta Precision Laboratories 590576 , Calgary, Alberta, Canada; 9 University of Pretoria 56410, Pretoria, Gauteng, South Africa; 10 Instituto de Biología Molecular y Celular de Rosario (IBR, CONICET-UNR), Rosario, Santa Fe, Argentina; 11 CWRU-Cleveland VAMC Center for Antimicrobial Resistance and Epidemiology (Case VA CARES), Cleveland, Ohio, USA; Universita degli Studi di Roma "La Sapienza", Rome, Italy

**Keywords:** metallo-beta-lactamase, IMP, xeruborbactam, carbapenemase

## Abstract

Metallo-β-lactamases (MBLs) of IMP type are not inhibited by currently commercialized β-lactamase inhibitors, including taniborbactam (TAN), which inhibits only NDM- and VIM-type enzymes. However, the development of xeruborbactam (XER), which additionally inhibits IMP enzymes, may provide effective drug combinations such as meropenem-XER (MEM-XER) against most MBL producers. Thirty-two IMP-producing clinical gram-negative isolates were tested for MEM-XER. Susceptibility testing of β-lactams with TAN or XER at 4 or 8 µg/mL was performed. Noticeably, MEM-XER remained ineffective against all IMP-producing *Pseudomonas aeruginosa* isolates. By contrast, supplementation with XER significantly lowered MEM MICs for several IMP-producing *Enterobacterales* isolates, except for isolates and recombinant *E. coli* strains producing IMP-6, IMP-10, IMP-14, and IMP-26. Interestingly, IMP-59 producers showed susceptibility to both TAN- and XER-based combinations, although IMP enzymes are not supposed to be inhibited by TAN. Determinations of 50% inhibitory concentration (IC_50_) values of XER showed values being >15-fold higher for IMP-6, IMP-10, IMP-14, and IMP-26 compared with IMP-1. Interestingly, the IC_50_ value of TAN for IMP-59 was found in the same range as that for NDM-1 (7 µM). Finally, structural analyses and molecular modeling simulations indicated that the Ser262Gly mutation in IMP-6 may alter the electronic properties of the active site, whereas the Phe residue in IMP-10 may exert a steric effect counteracting XER binding. Resistance to XER in IMP-6, IMP-10, IMP-14, and IMP-26 variants, conferring resistance to MEM-XER, might be considered a serious concern since MEM-XER will be supposed to be a salvage therapy for MBL-, and especially IMP-producing *Enterobacterales* infections.

## INTRODUCTION

The increasing trend in carbapenem resistance observed in gram-negative bacteria is mainly related to the dissemination and acquisition of carbapenemase-encoding genes (1). Among them, those encoding metallo-β-lactamases (MBLs) are the most problematic ones, since the production of MBLs leads to very limited treatment options. Indeed, MBLs of NDM-, VIM-, and IMP-types hydrolyze all β-lactams except monobactams, and their respective hydrolytic activities are not inactivated by currently commercialized β-lactamase inhibitors (2, 3). Hence, the most recently commercialized β-lactamase/β-lactamase inhibitor (BL/BLI) combinations such as ceftazidime-avibactam, ceftolozane-tazobactam, imipenem-relebactam, or meropenem-vaborbactam remain ineffective against MBL producers (4, 5). Since aztreonam can evade the hydrolysis of MBL, only this β-lactam alone or in combination with BLI can be effective against those MBL producers (6, 7).

However, the ongoing development of two novel boronate β-lactamase inhibitors, namely taniborbactam (TAN) (VNRX-5133) and xeruborbactam (XER) (QPX-7728), may shortly offer new perspectives in providing effective combinations against MBL producers (8, 9). Although TAN is supposed to be marked soon in combination with cefepime (FEP) and possesses remarkable inhibitory activity against NDM- and VIM-like enzymes (except against specific variants such as NDM-9, NDM-30, and VIM-83), it has no significant activity against IMP-type enzymes (10–13). This newly developed combination has now passed phase III of the clinical development, by showing superiority to meropenem (MEM) in urinary tract infections (14). By contrast, XER, which is in the early phase of clinical development in association with (MEM), significantly inhibits a wide range of IMP-type enzymes along with those of the VIM- and NDM-type β-lactamases (15, 16). This combination has already demonstrated promising *in vitro* activities when testing different collections of MBL producers, including IMP producers (17, 18). Another clinical study of phase I included a novel combination of XER with cefiderocol, which has also been initiated recently (Shionogi, personal communication).

IMP-1 was originally reported from *Pseudomonas aeruginosa* in 1988, then isolated in *Serratia marcescens* in Japan in 1991. Nowadays, more than 100 variants of IMP-like enzymes have been identified in many places in the world and in many gram-negative species, but still predominantly in Asia and Australia (19–21).

In the present study, we evaluated the *in vitro* activity of the promising combination under clinical development, MEM-XER, against a wide range of IMP-producing gram-negative clinical isolates to identify and determine potential resistance mechanisms to XER and the respective BL/BLI combination MEM-XER.

## RESULTS AND DISCUSSION

### Susceptibility testing with β-lactams in combination with XER and TAN for IMP-producing gram-negative clinical isolates

Most clinical isolates tested exhibited resistance to FEP, although showing variable MIC values for MEM (Table 1). MIC values of imipenem were found relatively low for *Enterobacterales* and relatively high for *P. aeruginosa* clinical isolates. Although TAN does not possess significant inhibitory activity against IMP enzymes, MICs of FEP-TAN were found to be lower than those of FEP alone when testing isolates producing IMP-6, IMP-26, IMP-34, and IMP-59 for *Enterobacterales*. When combining TAN with FEP or MEM, decreased MIC values were also observed for the SMART 1735 and AZ 1677 strains, when compared with the MICs of the respective β-lactams alone. Given that both isolates produced IMP-59 without concomitant ESBL enzyme production, this latter observation suggested that IMP-59 might be sensitive to the TAN inhibitory activity. It also suggested that the reduced MIC values observed for FEP-TAN in comparison to FEP alone for isolates producing IMP-6, IMP-26, and IMP-34 likely resulted from the co-production of an ESBL enzyme (CTX-M-like) being actually sensitive to TAN.

**TABLE 1:** Susceptibility testing of IMP-producing gram-negative isolates for xeruborbactam- and taniborbactam-based combinations^*a*^

ID strain	Strain	Accession no. of whole genome				Minimal inhibitory concentrations (µg/mL)^*c*^								
			ST	Country of isolation / year	Acquired β-lactamase (s)^*b*^	FEP	FEP-TAN	FEP-XER	IPM	IPM-TAN	IPM-XER	MEM	MEM-TAN	MEM-XER 8
QC 1	*Escherichia coli* NCTC 13353	NA	NA	NA	CTX-M-15, OXA-1	256	0.25	≤0.125	≤0.25	≤0.125	≤0.125	≤0.25	≤0.06	≤0.125
QC 2	*Klebsiella pneumoniae* ATCC BAA-2814	NA	NA	NA	**KPC-3**, SHV-11, TEM-1	>256	4	1	16	≤0.125	≤0.125	64	0.25	≤0.125
QC 3	*Escherichia coli* ATCC 25922	NA	NA	NA	-	≤0.25	≤0.125	≤0.125	≤0.25	≤0.125	≤0.125	≤0.25	≤0.06	≤0.125
QC 4	*P. aeruginosa* ATCC 27853	NA	NA	NA	-	1	1	1	1	1	1	0.5	0.5	0.25
E. coli 7 IMP	*Escherichia coli*	DRR065579	443	India 2008	**IMP-1**, CTX-M-15, OXA-10	>256	>128	8	0.5	0.5	≤0.125	1	1	≤0.06
SMART 958	*Citrobacter freundii*	DRR065635	100	Brazil 2013	**IMP-1,** OXA-142	32	32	0.25	0.5	0.5	≤0.125	4	4	≤0.06
SMART 1133	*Klebsiella pneumoniae*	DRR065640	37	Japan 2013	**IMP-1**, CTX-M-2	>256	>128	128	128	128	64	64	64	16
Kp 5 IMP	*Klebsiella pneumoniae*	DRR065578	478	Australia 2009	**IMP-4,** CTX-M-15, OXA-1, TEM-1	256	4	1	≤0.25	0.25	≤0.125	0.5	0.5	≤0.06
SMART 315	*C. freundii*	DRR065598	98	Australia 2011	**IMP-4**, CTX-M-9	16	16	0.5	≤0.25	0.25	≤0.125	2	2	≤0.06
SMART 1234	*Enterobacter hormaechei subsp. steigerwaltii*	SRR3110104	133	Australia 2014	**IMP-4**, SHV-12, OXA-1, TEM-1	8	8	0.25	≤0.25	≤0.125	≤0.125	0.5	0.5	≤0.06
SMART 1260	*Enterobacter hormaechei subsp. oharae*	SRR3110105	108	Australia 2014	**IMP-4,** LAP-2, OXA-1, TEM-1	32	32	2	≤0.25	0.25	≤0.125	1	1	0.25
SMART 1131	*Klebsiella pneumoniae*	DRR065638	37	Japan 2013	**IMP-6,** CTX-M-2	64	1	1	≤0.25	≤0.125	≤0.125	8	8	2
SMART 1132	*Escherichia coli*	DRR065639	131	Japan 2013	**IMP-6,** CTX-M-2	128	1	1	≤0.25	≤0.125	≤0.125	4	4	1
SMART 1137	*Klebsiella pneumoniae*	DRR065644	14	Japan 2013	**IMP-6**, CTX-M-2	>256	>256	>256	2	2	2	128	128	128
SMART 271	*Enterobacter hormaechei (cluster III*)	SRR2960061	78	Taiwan 2010	**IMP-8,** SHV-12, TEM-1	64	64	16	0.5	0.5	≤0.125	0.5	0.5	≤0.06
SMART 398	*Enterobacter hormaechei subsp. steigerwaltii*	SRR2960073	204	Taiwan 2011	**IMP-8,** LAP-2, TEM-1	128	128	8	0.5	0.5	≤0.125	0.5	0.5	≤0.06
SMART 2558	*Serratia marcescens*	NA	325	Taiwan 2016	**IMP-8,** CTX-M-3	32	32	0.5	≤0.25	0.25	≤0.125	0.25	0.25	≤0.06
SM1	*Serratia marcescens*	NA	NA	France	**IMP-11**	4	2	2	≤0.25	0.25	≤0.125	2	2	≤0.125
SMART 409	*Enterobacter xiangfangensis*	SRR2960075	182	Spain 2011	**IMP-13,** OXA-10, OXA-2	4	4	0.5	≤0.25	≤0.125	≤0.125	≤0.125	≤0.06	≤0.06
SMART 640	*Escherichia coli*	DRR065622	131	Thailand 2012	**IMP-14,** CTX-M-27, OXA-10	128	128	64	0.5	0.5	0.5	4	4	4
SMART 651	*Klebsiella pneumoniae*	DRR065625	70	Thailand 2012	**IMP-14**, SCO-1, TEM-1	64	64	64	1	1	1	8	8	8
SMART 1276	*Enterobacter hormaechei subsp. steigerwaltii*	SRR3110248	93	Thailand 2014	**IMP-14,** CTX-M-15, OXA-10, TEM-1	>256	>128	>128	0.5	0.5	0.5	2	2	1
SMART 875	*Klebsiella pneumoniae*	DRR065632	20	Philippines 2013	**IMP-26,** CTX-M-15, TEM-1 like	64	1	≤0.125	≤0.25	≤0.125	≤0.125	0.125	≤0.06	≤0.06
SMART 882	*Klebsiella pneumoniae*	DRR065634	2289	Philippines 2013	**IMP-26**, CTX-M-15, OXA-1	256	32	8	≤0.25	0.25	≤0.125	4	4	1
SMART 878	*Klebsiella pneumoniae*	DRR065633	14	Philippines 2013	**IMP-26**, CTX-M-15, OXA-1, TEM-1 like	256	16	4	≤0.25	0.25	≤0.125	4	4	1
SMART 1134	*Klebsiella pneumoniae*	DRR065641	14	Japan 2013	**IMP-34**, CTX-M-2	256	32	4	0.5	0.5	≤0.125	16	16	8
SMART 1735	*Escherichia coli*	NA	357	Australia 2015	**IMP-59,** TEM-1	8	0.25	≤0.125	≤0.25	≤0.125	≤0.125	0.5	≤0.06	≤0.06
AZ 1677	*Escherichia coli*	NA	357	Australia 2016	**IMP-59,** TEM-1	8	0.5	0.25	≤0.25	≤0.125	≤0.125	0.5	≤0.06	≤0.06
PA10	*Pseudomonas aeruginosa*	NA	NA	France	**IMP-2**	>256	>128	>128	32	32	32	32	32	32
CC02-010	*Pseudomonas aeruginosa*	NA	NA	NA	**IMP-7**	>256	>128	>128	128	128	128	256	>64	>128
C7	*Pseudomonas aeruginosa*	NA	NA	NA	**IMP-7**	>256	>128	>128	128	128	128	256	>64	>128
PA11	*Pseudomonas aeruginosa*	NA	NA	France 2013	**IMP-10**	>256	>128	>128	256	>128	>128	>256	>64	>128
PA12	*Pseudomonas aeruginosa*	NA	395	Switzerland 2023	**IMP-13**	128	128	128	8	8	8	16	16	16
PA13	*Pseudomonas aeruginosa*	NA	NA	France	**IMP-15**	128	128	128	256	>128	>128	64	64	64
PA14	*Pseudomonas aeruginosa*	NA	111	Switzerland 2020	**IMP-18**	256	>128	>128	128	128	128	32	32	32
PA15	*Pseudomonas aeruginosa*	NA	NA	France	**IMP-19**	>256	>128	>128	256	>128	>128	64	64	64
PA16	*Pseudomonas aeruginosa*	NA	NA	France	**IMP-29**	16	16	16	16	16	16	32	32	16

^*a*^ NA, data not available.

^*b*^ -, no acquired β-lactamase; the corresponding carbapenemases variants are highlighted in bold.

^*c*^ FEP, cefepime; FEP-TAN, cefepime-taniborbactam; FEP-XER, cefepime-xeruborbactam; IPM, imipenem; IPM-TAN, imipenem-taniborbactam; IPM-XER, imipenem-xeruborbactam; MEM, meropenem; MEM-TAN, meropenem-taniborbactam; MEM-XER, meropenem-xeruborbactam. In those BL/BLI combinations, taniborbactam and xeruborbactam were used at a fixed concentration of 4 µg/mL, except for MEM-XER 8, where xeruborbactam was used at a fixed concentration of 8 µg/mL.

On the other hand, no significant decrease in terms of MIC values was observed for all BL/BLI combinations in comparison to the respective β-lactams alone in all IMP-producing *P. aeruginosa* species. The absence of significant XER activity in this species precludes the ability to draw any conclusions regarding the sensitivity to XER against IMP-7, IMP-10, IMP-15, IMP-18, IMP-19, and IMP-29 once produced by such clinical isolates. This result is consistent with the findings of a recent study conducted on recombinant *P. aeruginosa* strains, which demonstrated the ineffectiveness of XER in that species. This outcome may be attributable to an overproduction of their intrinsic efflux systems, particularly the MexAB-OprM pump (16, 22). Also, considering that we previously showed that IMP-15, IMP-18, and IMP-19 variants were actually sensitive to XER, although IMP-10 was resistant to XER (16), the most likely hypothesis appears to be that MEM-XER is quite inefficient against *P. aeruginosa* isolates because of efflux mechanisms counteracting the efficacy of XER.

The combination of XER with β-lactams showed a significantly decreased MIC value in the majority of IMP-producing *Enterobacterales* isolates. However, this observation was not consistent with isolates producing IMP-6, IMP-14, IMP-26, and IMP-34, where less than 2-fold dilutions were observed between the MIC values of MEM-XER and MEM alone. Hence, MIC values of MEM-XER for SMART 640, SMART 651, and SMART 1276 isolates, all producing IMP-14, were determined at 4, 8, and 1 μg/mL, respectively, being identical to the respective MIC values when testing MEM alone. The same observation was made between MICs of MEM and MEM-XER (Table 1) for IMP-6-producing isolates SMART 1131 and SMART 1132. Likewise, no difference was observed with the IMP-34 producer (both MICs being at 8 μg/mL). Finally, SMART 878 and SMART 882 isolates, both producing IMP-26, also displayed identical MIC values of MEM and MEM-XER (1 μg/mL). Those results suggested that the following IMP variants, namely IMP-6, IMP-7, IMP-10, IMP-14, IMP-26, and IMP-34, might be resistant to XER in addition to TAN and that the IMP-59 variant might be sensitive to both TAN and XER.

### Susceptibility testing with β-lactams in combination with XER and TAN for IMP-producing recombinant *E. coli* strains

Susceptibility testing using BL/BLI combinations against IMP-producing *E. coli* recombinant strains revealed some interesting features (Table 2). First, a significant number of IMP-like producers displayed variable MIC values for β-lactams, thus highlighting that specific amino-acid substitutions significantly influenced their respective hydrolysis spectrum, as suggested by previous studies (23–25). Hence, the IMP-6 (IMP-1 Ser262Gly)-producing recombinant *E. coli* strain exhibited higher MIC values for MEM but lower MIC values for FEP in comparison to IMP-1. Also, the IMP-10 (IMP-1 Val67Phe) and IMP-26 (IMP-4 Val67Phe) producers showed significantly elevated MIC values for FEP and carbapenems when compared with the IMP-1 or IMP-4 producers.

**TABLE 2:** Susceptibility testing with β-lactams in combination with xeruborbactam and taniborbactam for IMP-producing recombinant *E. coli^a^*

Strain (β-lactamase produced)	MBL variant	Amino acid substitution from the respective MBL variant	Minimal inhibitory concentrations (µg/mL)								
			FEP	FEP-TAN (4 µg/mL)	FEP-XER (4 µg/mL)	IPM	IPM-TAN (4 µg/mL)	IPM-XER (4 µg/mL)	MEM	MEM-TAN (4 µg/mL)	MEM-XER (8 µg/mL)
*E. coli* NCTC 13353	NA	-	256	0.25	≤0.125	≤0.25	≤0.125	≤0.125	≤0.25	≤0.06	≤0.125
*Klebsiella pneumoniae* ATCC BAA-2814	NA	-	>256	4	1	16	≤0.125	≤0.125	64	0.25	≤0.125
*E. coli* TOP10	NA	-	≤0.25	≤0.125	≤0.125	≤0.25	≤0.125	≤0.125	≤0.25	≤0.125	≤0.125
*E. coli* IMP-1	IMP-1-like	-	32	32	≤0.125	4	4	≤0.125	8	8	≤0.125
*E. coli* IMP-6		S262G	4	4	4	≤0.25	0.125	0.125	64	64	64
*E. coli* IMP-10		V67F	64	64	16	16	16	8	64	64	32
*E. coli* IMP-34		E126G	2	2	≤0.125	0.5	0.5	≤0.125	2	2	≤0.125
*E. coli* IMP-2	IMP-2-like	-	8	8	0.25	0.5	0.5	≤0.125	1	1	≤0.125
*E. coli* IMP-14		Multiples	64	64	64	1	1	1	4	4	4
*E. coli* IMP-14 Mutant 1		S65G	128	128	128	8	4	4	8	8	4
*E. coli* IMP-14 Mutant 2		H174N	64	64	64	2	2	1	4	4	4
*E. coli* IMP-14 Mutant 3		N178S	128	128	128	2	2	1	8	8	8
*E. coli* IMP-14 Mutant 4		D227Y	128	128	128	8	8	8	8	8	8
*E. coli* IMP-14 Mutant 5		Y233N	16	16	16	32	32	32	1	1	0.5
*E. coli* IMP-4	IMP-4 like	-	8	8	≤0.125	1	1	≤0.125	8	8	≤0.125
*E. coli* IMP-26		V67F	32	32	8	8	8	2	128	128	32
*E. coli* IMP-59		N233Y	8	1	0.25	0.5	≤0.125	≤0.125	8	0.5	≤0.125
*E. coli* IMP-7	IMP-7-like	-	2	2	≤0.125	0.25	0.25	≤0.125	2	2	≤0.125
*E. coli* NDM-1	NDM-1-like	-	4	0.25	≤0.125	2	≤0.125	≤0.125	2	≤0.125	≤0.125
*E. coli* NDM-1*		S262G	1	1	1	0.25	0.25	0.25	32	32	16

^*a*^ NA, not applicable; FEP, cefepime; IPM, imipenem; MEM, meropenem; TAN, taniborbactam; XER, xeruborbactam. Nomenclature is according to the BBL sequence numbering.

Combining β-lactams with XER resulted in significantly lower MIC values for the IMP-1, IMP-2, and IMP-4 producers, as expected. However, in line with results observed with clinical isolates, supplementation with XER (either at concentrations of 4 or 8 µg/mL) did not significantly decrease the respective MIC values against IMP-6, IMP-10, IMP-14, and IMP-26 producers. Such observations had already been made for IMP-10 and IMP-26 producers in previous studies (16, 17). Conversely, significant reductions in the MIC values were observed for IMP-34 and IMP-7 producers, leading to low MIC values for XER-based combinations.

On the other hand, and as expected, the different combinations of β-lactams with TAN did not result in significantly lower MIC values for all IMP producers, with an exception observed with the IMP-59 producer.

### Relative inhibitory activities of XER and TAN against IMP-like enzymes

In order to better evaluate the relative capacities of XER and TAN to inhibit IMP-like enzymes, the respective 50% inhibitory concentration (IC_50_) values were determined. As shown in Table 3, the lack of significant inhibition of IMP-6, IMP-10, IMP-14, and IMP-26 ß-lactamases by XER and by TAN was confirmed. Indeed, the IC_50_ values of XER for the IMP-1-like variants, IMP-6, and IMP-10, were found to be 180 and 73 µM, respectively, being 30-fold higher than for IMP-1. As for the IMP-2-like variants, IMP-14 exhibited an IC_50_ value that was almost 100-fold higher than that of IMP-19. The relatively high IC_50_ of XER against IMP-26 was in the same range as found against IMP-10, with both enzymes exhibiting the same substitution at position 67 (Val67Phe), in comparison to IMP-4 and IMP-1, respectively. As anticipated, elevated IC_50_ values of TAN (>250 µM) were observed for the aforementioned variants.

**TABLE 3:** Determination of the 50% inhibitory concentrations (IC_50_) of xeruborbactam according to different subclasses and variants of metallo-β-lactamases, in comparison to taniborbactam^*a*^

MBL enzyme	Ambler class	MBL variant	IC_50_ xeruborbactam (µM)	IC_50_ taniborbactam (µM)
		NDM-1^*b*^	4.3	3.0
NDM-like	B1	NDM-1 S262G	>200	>250
IMP-like	IMP-1- like	IMP-1^*b*^	2.7	>250
		IMP-6	180	>250
		IMP-10^*b*^	73	>250
	IMP-2-like	IMP-19^*b*^	7.5	>250
		IMP-14	>250 (680)	>250
		IMP-14 S65G	140	>250
		IMP-14 H174N	182	>250
		IMP-14 N178S	180	>250
		IMP-14 D227Y	250	204
		IMP-14 N233Y	210	>250
	IMP-4-like	IMP-4^*b*^	14.7	>250
		IMP-26	77	>250
		IMP-59	10.3	7.0

^*a*^ Nomenclature is according to the BBL sequence numbering.

^*b*^ For those enzymes, results were reported from a previous study (16).

Surprisingly, a low IC_50_ value of TAN was found against IMP-59, a value in the same range as the one found against NDM-1, whereas the IC_50_ of XER against IMP-59 remained in the same range as that found with IMP-4.

To summarize, IMP-6, IMP-10, IMP-14, and IMP-26 were defined as resistant to XER, and consequently to the two MBL inhibitors supposed to be clinically available in the near future, and IMP-59 was the only IMP variant identified as being sensitive to both TAN and XER.

### Structural features of the IMP variants resistant to XER

**Fig 1:**
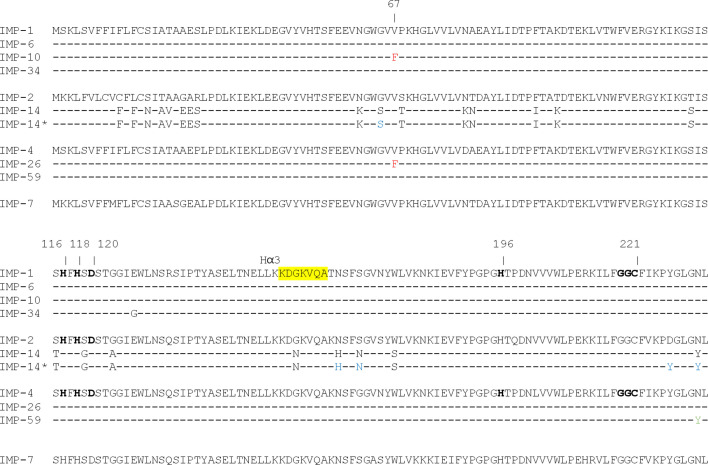
Sequences alignments of IMP-like enzymes. Nomenclature is according to the BBL sequence numbering. (-), identical residue compared to IMP-1 sequence; The omega loop region of IMP-like enzymes is yellow-shaded. Residues in bold are zinc ligands conserved in the subclass B1 MBL enzymes. Residues in red are those identified for xeruborbactam resistance. Residue in green is identified for taniborbactam sensitivity. Residues in blue are those tested for reversing xeruborbactam resistance in IMP-14.

**Fig 2:**
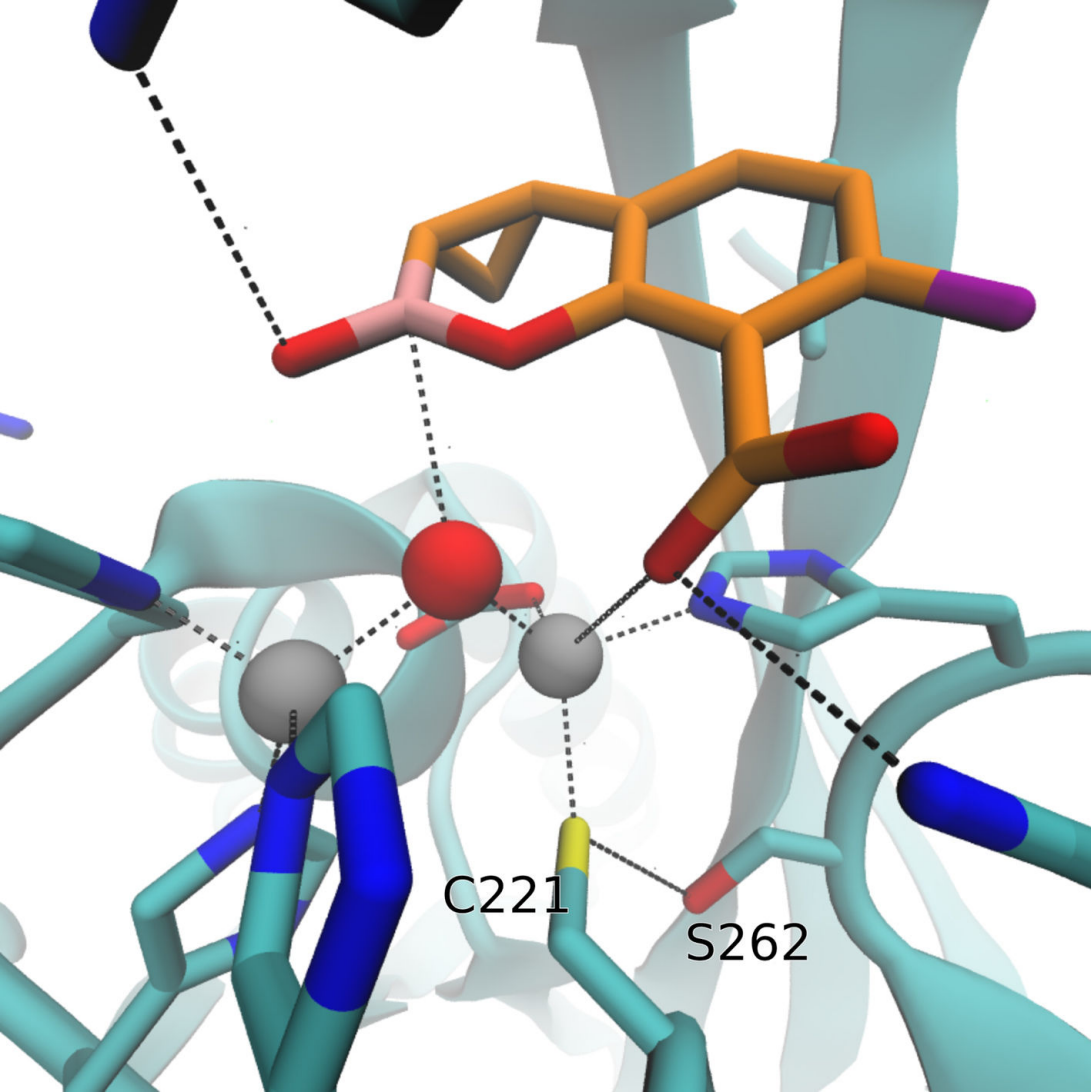
Docking simulation pose for XER with IMP-1 showing the position of Ser262 below the active site. XER and selected residues of the active site are depicted in licorice. C atoms of XER are colored in orange, C atoms of IMP-1 in cyan, O atoms in red, B atom in pink, F atom in purple, N atoms in blue, and S atom in yellow. Zn ions are shown as gray spheres.

**Fig 3:**
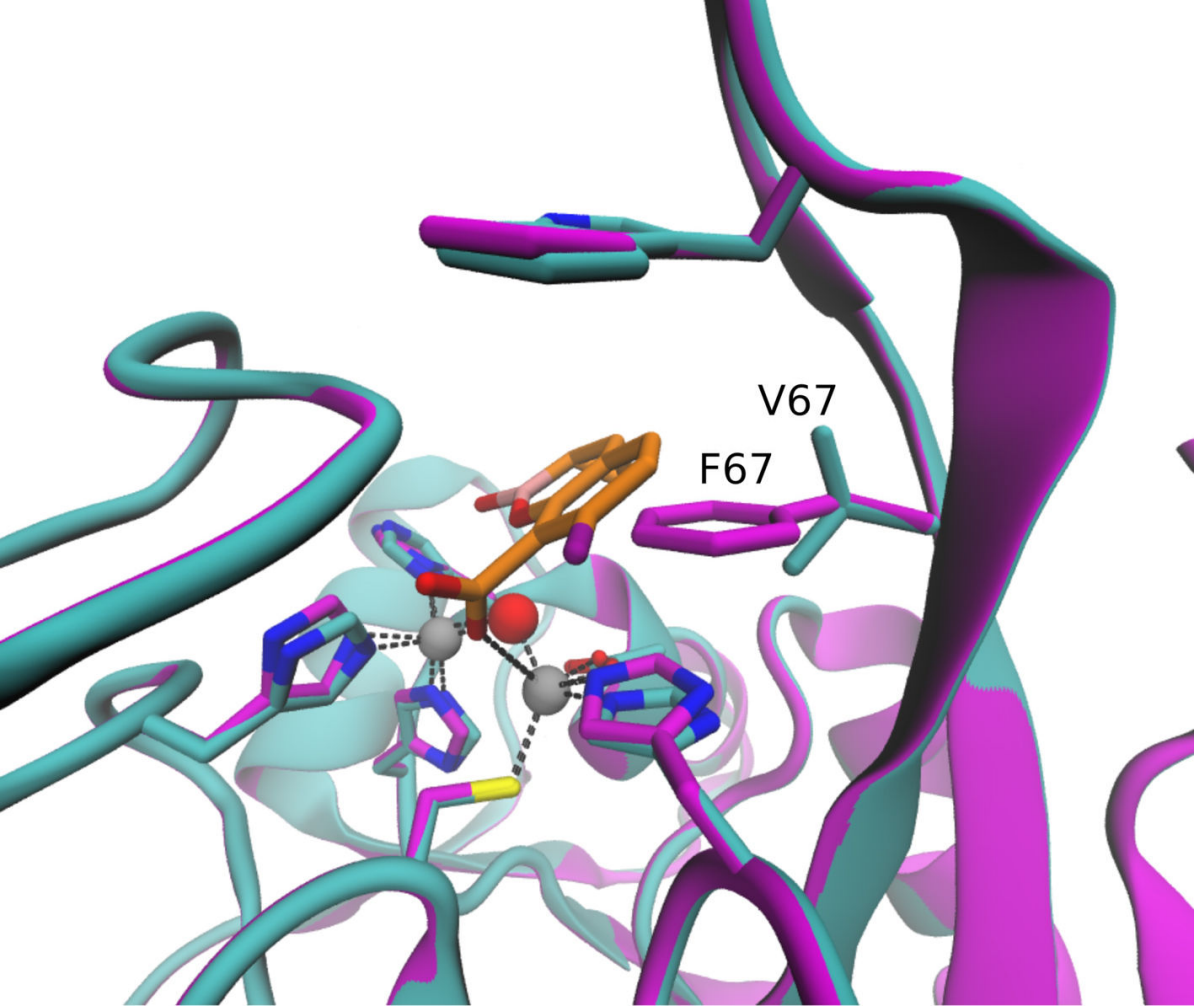
Docking simulation results of XER in IMP-1 (cyan), aligned with the protein IMP-10 (magenta). XER and selected residues are depicted in licorice. C atoms of XER are colored in orange, C atoms of IMP-1 in cyan, C atoms of IMP-10 in magenta, O atoms in red, B atoms in pink, F atoms in purple, N atoms in blue, and S atoms in yellow. Zn ions are shown as gray spheres.

IMP-6 and IMP-10 differ from IMP-1 by only a single amino-acid substitution at positions 262 (Ser262Gly) or 67 (Val67Phe), respectively (Fig. 1). IMP-26 differs from IMP-4 by the same Val67Phe substitution. All those IMP variants were found resistant to the inhibitory action of XER. Structural analysis including docking simulations showed that Ser262Gly substitution in IMP-1 may alter the electronic properties of the active site, since the Ser residue in the wild-type protein is coordinated to the active site residue Cys221 and therefore could affect the binding of XER, as illustrated in Fig. 2. Other studies also propose that this substitution disrupts a hydrogen-bonding interaction with the base of the L3 loop, altering its conformation and its substrate profile (26, 27). This position has been shown to be a mutational hotspot also in directed evolution experiments of the model enzyme BcII by tuning the flexibility of loop L3. Further structural analysis together with docking studies (28, 29) suggests that the Val67Phe substitution identified in IMP-10 and IMP-26 may result in a steric effect, which has the potential to impede the positioning of XER. Docking on these proteins failed to produce productive poses, which can be explained when aligning these proteins with the docking results of IMP-1, as illustrated in Fig. 3.

A comparison of the protein sequences of NDM-1 and IMP-1 revealed that both enzymes shared a serine at position 262. Therefore, site-directed mutagenesis was performed to generate an NDM-1 variant, namely NDM-1 Ser262Gly, to eventually evaluate its sensitivity to XER by analogy to the results we obtained with IMP-6. Interestingly, supplementation of β-lactams with XER did not result in decreased MIC values when testing the recombinant *E. coli*-producing NDM-1 Ser262Gly, whereas its ability to confer resistance to β-lactams was not altered (Table 2). Determination of the corresponding IC_50_ for XER confirmed the resistance of this NDM-1 mutant to XER (>200 µM) (Table 3). This confirms the role of position 262 as a mutational hotspot in the second coordination sphere of the active site that can elicit a large impact on different MBLs, despite being phylogenetically distant.

### IMP-14, an IMP-2 variant showing resistance to XER

IMP-14 differs from IMP-2 by several amino-acid substitutions, both enzymes sharing ca. 90% amino acid identity. IMP-14 was found to be resistant to XER, according to the high measured IC_50_ value (650 µM). By aligning the protein sequence of IMP-14 with those of IMP-2 and other IMP variants that are sensitive to XER, a series of putative amino acid candidates sustaining the resistance feature could be identified, namely Ser65Gly, His174Asn, Asn177Ser, Asp227Tyr, and Asn235Tyr. Then, site-directed mutagenesis experiments were performed to separately reverse those five selected substitutions, generating five distinct mutated IMP-14 enzymes. As a result, none of the generated mutants recovered complete sensitivity to XER upon measurement of the respective IC_50_ values, taking IMP-1 as a reference. Nevertheless, each generated substitution led to a partial recovery of sensitivity to XER, as evidenced by the reduced IC_50_ values (ca. 3-fold) measured for all those IMP-14 mutants in comparison to the value obtained for IMP-14 (Table 3). It therefore suggests that the resistance to XER of IMP-14 is related to several of those substitutions, if not all. In contrast to the findings in IMP-6, which discloses a mutational hotspot, the impact of these mutations is context-dependent, revealing a strong epistatic interaction among them.

**Fig 4:**
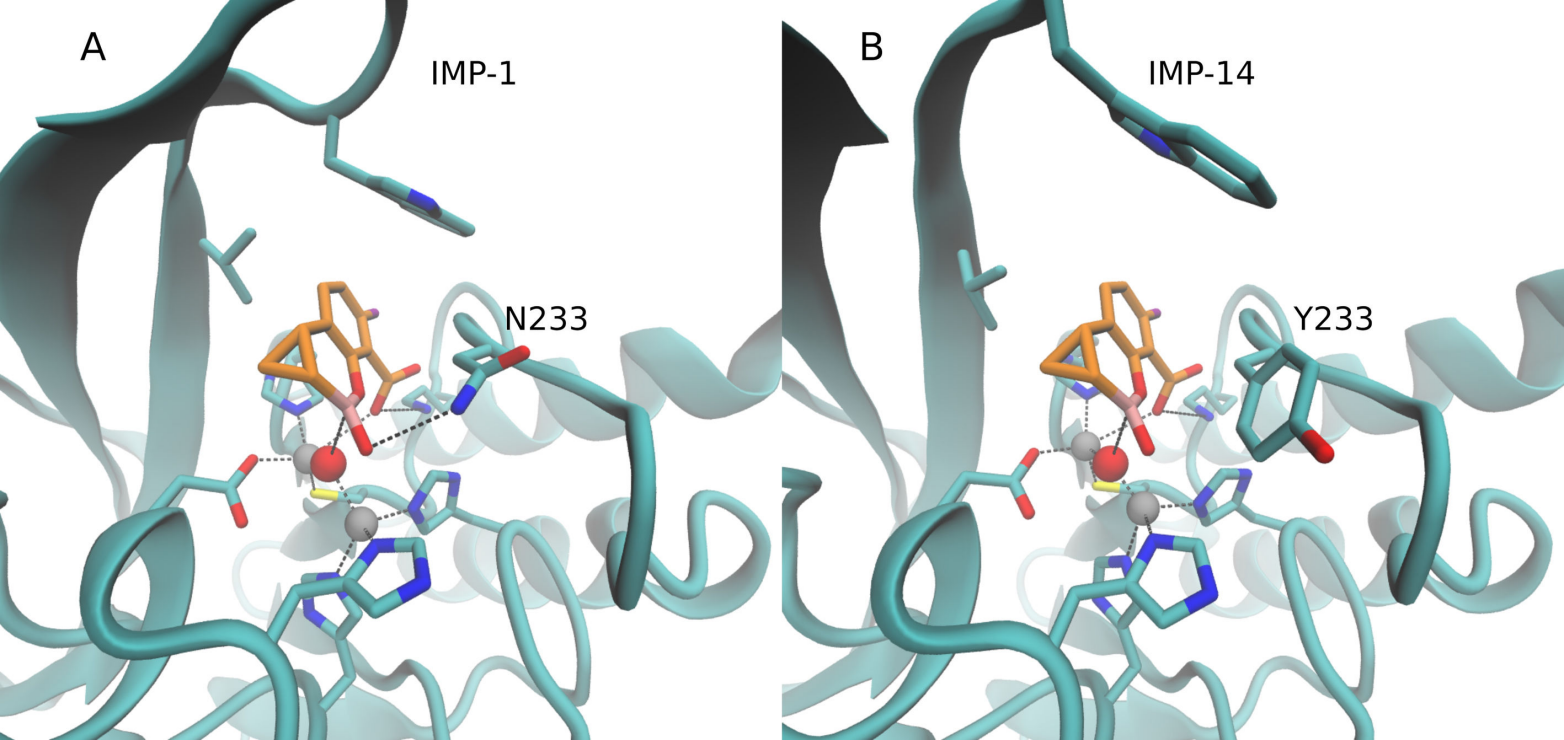
Docking simulation results for XER with IMP-14 (**A**) in comparison to IMP-1 (**B**). XER and selected residues are depicted in licorice. C atoms of XER are colored in orange, C atoms of IMP-1/IMP-59 in cyan, O atoms in red, B atoms in pink, F atoms in purple, and N atoms in blue. The Zn ions and the O atom of the nucleophilic OH^-^ are shown as gray and red spheres, respectively.

Structural analysis of IMP-14 revealed that Asn233Tyr could reduce the interaction energy with XER by losing the possibility to form a hydrogen bridge (Fig. 4). However, this cannot fully account for the lack of sensitivity to XER, as IMP-59, which is sensitive to XER, also harbors the same substitution. Consequently, it can be deduced that other mutations present in IMP-14 might be playing a role in decreasing interaction energy. The Asp227Tyr mutation was also generated in order to assess whether an impact of XER sensitivity could potentially be observed when two Tyr residues are located at positions 227 and 233, respectively, considering results observed with IMP-59 (Fig. S1). This substitution led to a slight decrease in resistance to TAN, being, however, too weak to be clearly significant, as indicated by the IC_50_ value (204 µM). No decrease in MIC values was observed for the combination of β-lactams with TAN when testing the recombinant *E. coli* producing IMP-14 Asp227Tyr (Table 2).

### IMP-59, a specific IMP-4 variant sensitive to XER and TAN

**Fig 5:**
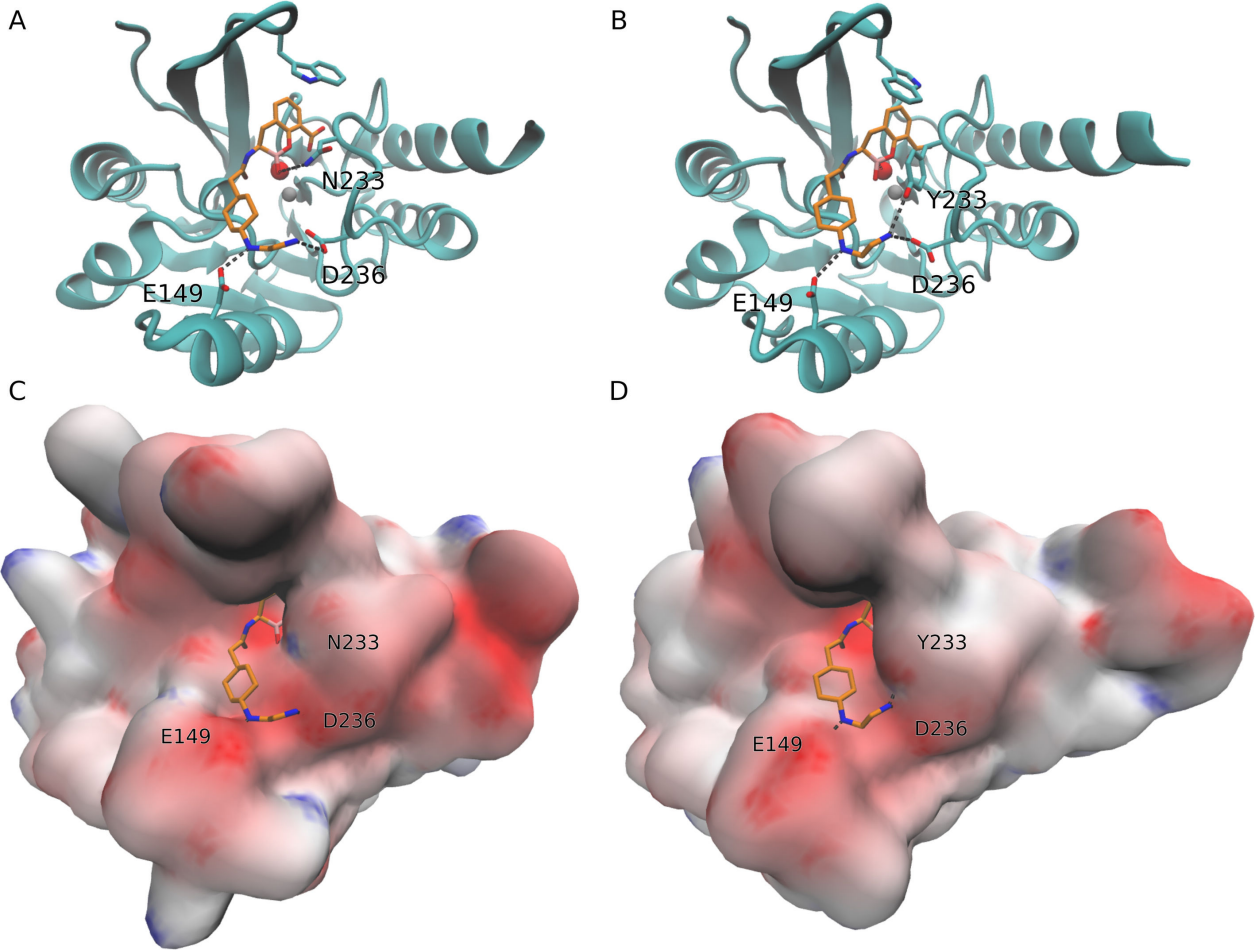
Docking simulation results for taniborbactam with IMP-1 (**A**) and IMP-59 (**B**) and electrostatic potential surfaces for IMP-1 (**C**) and IMP-59 (**D**). For visual clarity, only the side chains of residues of the active site and those that interact with the N-(2-aminoethyl)cyclohexylamine moiety of TAN are shown, and hydrogen atoms are omitted. Negative, neutral, and positive potentials are colored as red, white, and blue, respectively, on a scale from −8 to 8 k_B_T/e_c_. TAN and selected residues are depicted in licorice. C atoms of TAN are colored in orange, C atoms of IMP-1/IMP-59 in cyan, O atoms in red, and N atoms in blue. The Zn ions and the O atom of the nucleophilic OH^-^ are shown as gray and red spheres, respectively.

As shown in Fig. 1, IMP-59 differs from IMP-4 by a single amino-acid substitution at position 233 (Asn233Tyr). Interestingly, high sensitivities to both TAN and XER were observed for IMP-59 (Tables 2 and 3). Those observations provide strong evidence that the Asn233Tyr substitution plays a crucial role in the sensitivity to TAN of IMP-4-like enzymes. The structural analysis suggested that the amino acid Tyr at position 233 might increase the interaction energy by interacting with the amino group in TAN (Fig. 5A and B). However, this cannot fully explain the sensitivity to TAN, since IMP-14 harbors the same Tyr residue while showing resistance to TAN. It can therefore be hypothesized that both Tyr residues at positions 233 and 277 are necessary to get sensitivity to TAN, a hypothesis that is in line with previous results observed with the IMP-14 Asp227Tyr mutant, showing a decreased IC_50_ value of TAN in comparison to IMP-14.

It is noteworthy that a previous study has already identified the absence of negative electrostatic surface as the primary factor contributing to TAN resistance among IMP-like β-lactamases, in comparison to other MBLs such as NDM or VIM-like enzymes (13). The present study suggests that the Asn233Tyr substitution in the sequence of IMP-4-like enzymes can enhance the negative electrostatic surface on IMP-59, thereby improving its binding affinity to TAN (Fig. 5C and D). This suggests that substitutions with more subtle electrostatic effects can have a large impact on the inhibitory profile of TAN, highlighting the need to study the profile of the different allelic variants.

### Conclusion

Several interesting features were evidenced throughout the present study. First, the novel combination under development, MEM-XER, was shown to be ineffective against the tested IMP-producing *P. aeruginosa* clinical isolates, in contrast to what was observed when testing IMP-producing *Enterobacterales*. This observation could be attributable, at least in part, to the efflux system MexAB-OprM produced by *P. aeruginosa* isolates, as previously postulated when testing recombinant MBL-producing *P. aeruginosa* strains (16, 22). Second, this study identified additional IMP variants—namely IMP-6 and IMP-14—as being resistant to XER, and therefore potential sources of resistance to the MEM-XER combination, in addition to the previously reported IMP-10 and IMP-26 (16, 17). Furthermore, this study provided a more in-depth analysis of the resistance mechanisms of these IMP variants to XER and identified the first IMP-type enzyme demonstrating sensitivity to TAN, namely IMP-59. With the exception of IMP-14, these XER-resistant variants exhibited a single amino acid substitution compared with IMP-1 or IMP-4, suggesting that such mutation could potentially arise under selective pressure. Notably, the IMP-10 and IMP-26 variants have been documented in a variety of gram-negative bacteria worldwide, including Japan, South Korea, China, Reunion Island, Brazil, and the Philippines (30–36). On the other hand, IMP-6 has been identified in both *Enterobacterales* and *P. aeruginosa* and is widely distributed throughout Asia, being one of the most prevalent carbapenemase enzymes in Japan (37, 38), South Korea (39), and China (40). The substitution of Ser262Gly appears to enhance flexibility within the loop via the formation of a hydrogen bond with Pro68. Notably, this modification results in a distinct phenotype pattern in IMP-6-producing *Enterobacterales*, characterized as imipenem susceptibility and meropenem resistance (41, 42). A recent paper has also reported a decrease in susceptibility to novel penems, such as tebipenem, in recombinant *E. coli* strains producing IMP-6 and IMP-10, compared with those producing IMP-1 (43).

The present study demonstrates that the factors altering the sensitivities of β-lactamase inhibitors for certain IMP variants, such as IMP-14 to XER or IMP-59 to TAN, are intricate and cannot be wholly explained by single residue substitutions. Rather, they are the result of an interaction between multiple mutations. Nevertheless, it is worth highlighting that IMP-14 has so far only been reported in Thailand, in both *K. pneumoniae* and *P. aeruginosa* (44, 45). As for IMP-59, which is currently the only known IMP variant found to be sensitive to TAN, this variant has been identified in *E. coli* clinical isolates in Australia (46). Consequently, IMP-6, IMP-10, IMP-14, and IMP-26 are classified as resistant to XER and TAN, whereas IMP-59 is sensitive to TAN and XER.

Finally, the development of XER may offer new therapeutic options for MBL producers, particularly those producing IMP enzymes. However, the fact that the IMP-6, IMP-10, IMP-14, and IMP-26 variants are resistant to XER might be a serious problem, since XER is meant to be a treatment for MBL- and especially IMP-producing *Enterobacterales* infections, and the report of inefficacy of the novel combination, MEM-XER, against IMP-producing *P. aeruginosa* isolates is a major concern, given that IMP-type enzymes are primarily identified in *P. aeruginosa* species.

## MATERIALS AND METHODS

### Bacterial isolates

A collection of 32 IMP-producing clinical gram-negative isolates comprising a wide range of IMP variants (IMP-1, 4, 5, 6, 7, 8, 10, 11, 12, 13, 14, 15, 18, 19, 26, 34, 59) and partly collected from a global surveillance program, namely the Merck Study for Monitoring Antimicrobial Resistance Trends (SMART) (2008–2014) were included in the study (35, 47–51). Most isolates and their respective β-lactamase content were characterized by whole-genome sequencing.

### Cloning experiments and site-directed mutagenesis

Genes encoding different acquired IMP-like enzymes suspected to be resistant to XER (*bla*_IMP-6_*, bla*_IMP-7_*, bla*_IMP-10_*, bla*_IMP-14_*, bla*_IMP-26_*, bla*_IMP-34_*,* and *bla*_IMP-59_) were amplified by PCR, and corresponding amplicons were cloned into plasmid pUCP24 and expressed in *E. coli* TOP10. The following recombinant *E. coli* strains producing IMP-1, IMP-4, IMP-19, and NDM-1 were used from a previous study (16). Therefore, site-directed mutagenesis was performed using the Q5 Site-Directed Mutagenesis kit (ref. E0554S, New England Biolabs, Ipswich, MA) to obtain mutants of NDM-1 and IMP-14 in order to test the impact of specific amino-acid substitutions with respect to XER sensitivity. For that purpose, a list of primers was designed and provided as Table S1. The VIM-1 protein sequence already exhibits a glycine at position 262, rather than a serine. Hence, generating mutants to evaluate the impact of Ser262Gly on VIM-like enzymes was not deemed appropriate. The nomenclature used in this study was according to the BBL sequence numbering (52).

### Susceptibility testing

To assess the inhibitory potency of XER, susceptibility testing was performed by broth microdilution method, determining the minimal inhibitory concentrations (MICs) of different antibiotics or antibiotic plus β-lactamase inhibitor combinations, including cefepime (FEP), imipenem (IPM), meropenem (MEM), and their combinations with XER (FEP-XER, IPM-XER, and MEM-XER). All data obtained with XER-based combinations were compared with data with TAN-based combinations, FEP-TAN, IPM-TAN, and MEM-TAN (16). FEP was purchased from Sigma-Aldrich (Saint-Louis, USA), whereas MEM was from HuiChem (Shanghai, China). TAN hydrochloride (HY-109124A) and XER disodium (HY-136072) were purchased from MedChem Express (Luzern, Switzerland) and were used at fixed concentration at 4 µg/mL for all BL/BLI combinations, except MEM-XER, which has been evaluated using a fixed concentration of XER at 8 µg/mL, in order to be in line with previous works on MEM-XER combinations (15–18). MICs were determined in triplicate in cation-adjusted Mueller-Hinton (MH) broth (Bio-Rad, Marnes-la-Coquette, France) according to the CLSI guidelines (53). The results were interpreted according to the decrease in MIC value for the same antibiotic or combination of the corresponding recombinant strain producing the MBL enzyme. The reference strains *E. coli* ATCC 25922, *E. coli* NCTC 13353, *K. pneumoniae* ATCC BAA-2814, as well as the recombinant *E. coli* TOP10 (pIMP-1) were used as controls for all testing according to CLSI and previous works (15–18, 53). In a recent phase I clinical evaluation, XER was combined with cefiderocol. However, as this latter β-lactam substrate is known to be poorly hydrolyzed by IMP enzymes, it was not considered for the evaluation of XER inhibitory activity in this study. Instead, carbapenems were used as a substrate to evaluate the β-lactamase IMP activity in the presence or absence of XER and TAN, as it was deemed more specific for the IMP carbapenemase activity.

### β-lactamase activities

Cultures of *E. coli* TOP10 harboring recombinant plasmids and therefore producing the different IMP-like tested were grown overnight at 37°C in 50 mL of lysogeny broth (LB) with gentamicin (50 µg/mL). The bacterial suspension was pelleted, resuspended in 10 mL of 100 mM phosphate buffer (pH 7) supplemented with ZnSO_4_ (5  µM) for *E. coli* TOP10 harboring IMPs and then sonicated using a Vibra Cell 75186 sonicator (Thermo Fisher Scientific), followed by centrifugation for 1 h at 11,000 × *g* and 4°C. The presence of the β-lactamase was monitored using nitrocefin (200  µM). Kinetic measurements were performed at room temperature in 100 mM sodium phosphate (pH 7.0) supplemented with ZnSO_4_ (5  µM) for IMPs using a UV/visible ULTROSPEC 2100 pro spectrophotometer (Amersham Biosciences, Buckinghamshire, UK). The wavelength and absorption coefficient of cephalothin (262 nm/Ʌ ɛ = −7960 M^−1^.cm^−1^) were used for all IMPs, and IC_50_ was determined in triplicate for TAN and XER. Various concentrations of these inhibitors were pre-incubated with the crude extract of the enzyme for 3 min at room temperature to determine the concentrations that reduced the hydrolysis rate of 100 µM of cephalothin by 50% (54). To provide comparable results on the potency of β-lactamase inhibitor activity, each concentration of lysate was adjusted to hydrolyze cephalothin with relatively similar rates, defined by the concentration of lysate hydrolyzing cephalothin with a slope between 0.06 and 0.1 OD/min.

### Docking simulations

All docking simulations were performed using Autodock4 (55) with a grid map of 70 × 70 × 70 points with a grid spacing of 0.375 Å centered on the oxygen atom of the hydroxyl ion in the catalytic site. Charges for the Zn^2+^ and hydroxyl ions present in the catalytic center of the MBLs were obtained from the literature (56). The structures for IMP-1 were taken from the Protein Data Bank with code 7XHW (38). The structure for IMP-10 was built *in silico* by making the single V67F mutation on IMP-1 using Tleap (57). The structures for IMP-14 and IMP-59 were built *in silico* using the I-TASSER server (58). Structures of XER and TAN were shown in the supplementary materials (Fig. S2) and obtained from PubChem and optimized using Gaussian09 (59).

### Electrostatic surface calculations

Electrostatic potential surfaces were calculated using APBS software (60). Figures showing surface visualization were produced using VMD software (61), with quicksurf drawing method with a radius scale of 0.8, a density isovalue of 0.9, and a color scale data range from −10 to 10 kBT/ec.

## Data Availability

All data from this study can be made available upon request, without limitation in time.

## SUPPLEMENTAL MATERIAL

Fig. S1 aac.00297-25-s0001.pdf Comparison of the structures of the IMP variants showing the mutations in the L10 loop.

Fig. S2 aac.00297-25-s0002.pdf Structures of Taniborbactam and Xeruborbactam β-lactamase inhibitors.

Table S1 aac.00297-25-s0003.pdf Primers used for site-directed mutagenesis experiments.

ASM does not own the copyrights to Supplemental Material that may be linked to, or accessed through, an article. The authors have granted ASM a non-exclusive, world-wide license to publish the Supplemental Material files. Please contact the corresponding author directly for reuse.
