# Supplementary material for: The emerging concern of IMP variants being resistant to the only IMP-type metallo-β-lactamase inhibitor, xeruborbactam
Source: Antimicrob Agents Chemother. 2025 Jun 9;69(7):e00297-25. doi: 10.1128/aac.00297-25 (PMC12217461; doi:10.1128/aac.00297-25)
Supplement: Fig. S1 — Comparison of the structures of the IMP variants showing the mutations in the L10 loop. [file aac.00297-25-s0001.pdf]

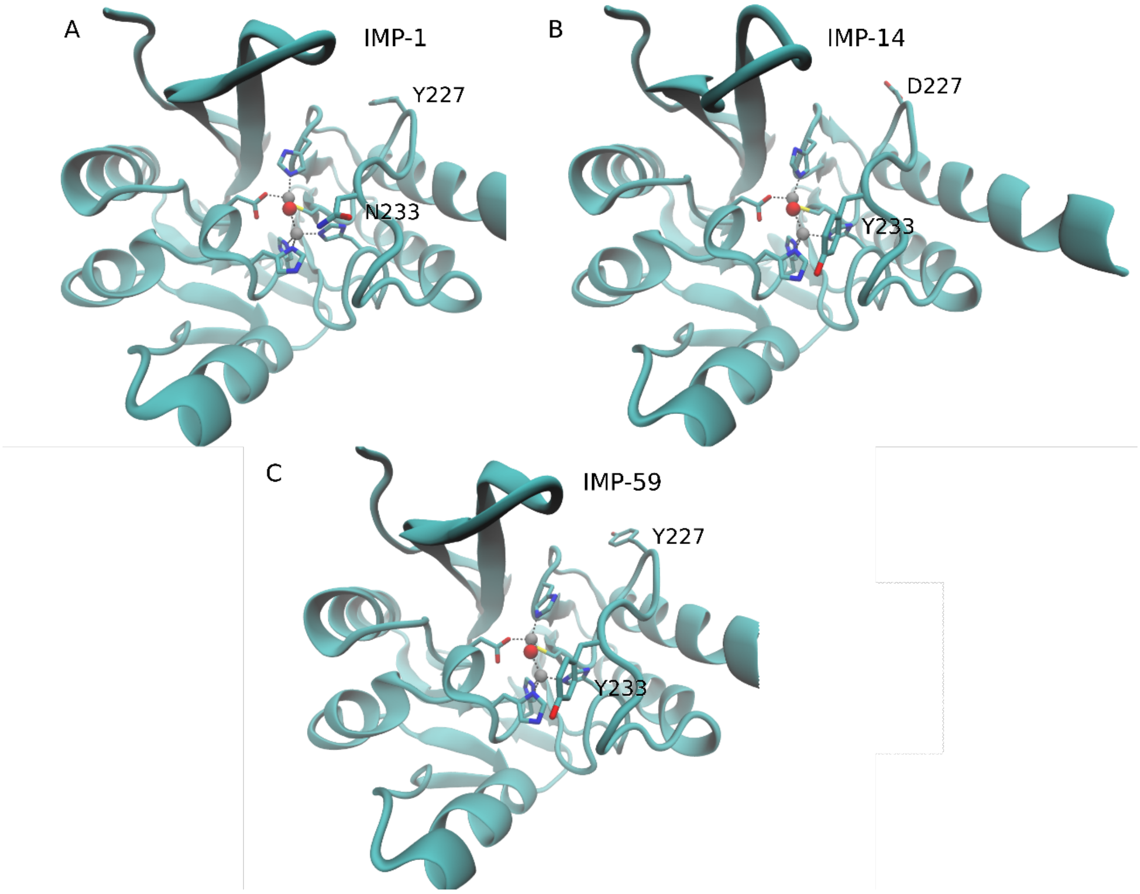

Figure S1. Comparison of the structures of the IMP variants showing the mutations in the L10 loop. IMP-1 (A), IMP-14 (B) and IMP-59 (C)
