# Supplementary figures and images for: The emerging concern of IMP variants being resistant to the only IMP-type metallo-β-lactamase inhibitor, xeruborbactam
Source: Antimicrob Agents Chemother. 2025 Jun 9;69(7):e00297-25. doi: 10.1128/aac.00297-25 (PMC12217461; doi:10.1128/aac.00297-25)

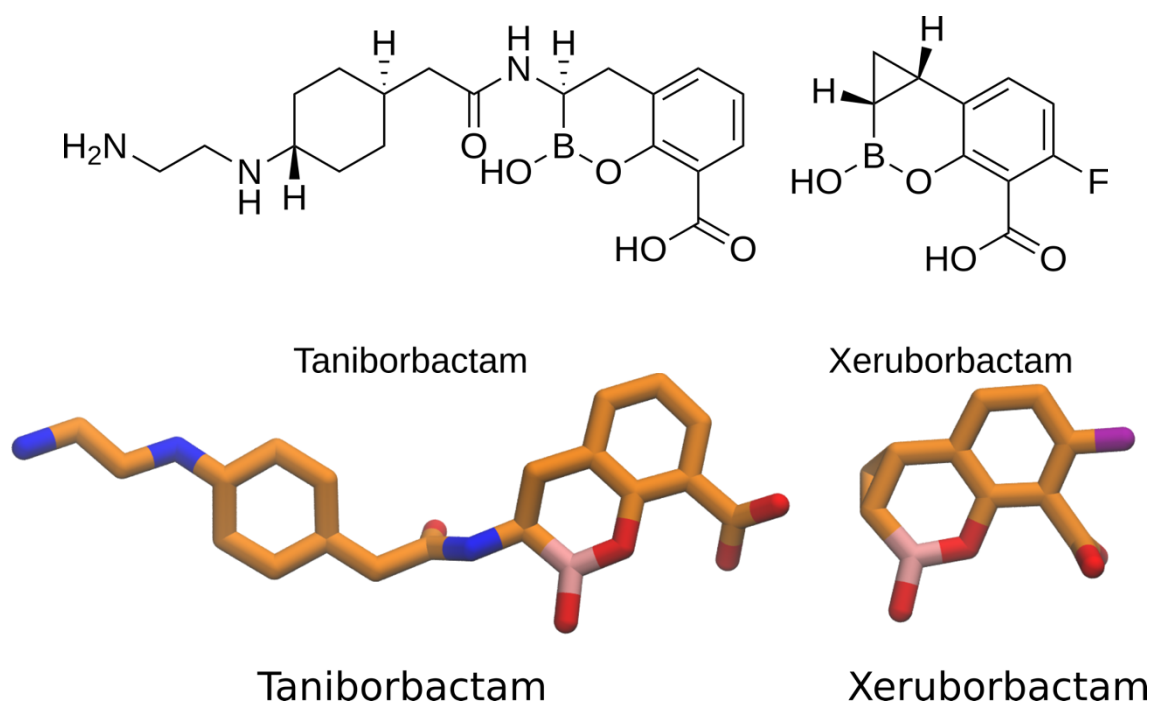

**Figure S2.** Structures of Taniborbactam and Xeruborbactam  $\beta$ -lactamase inhibitors.

Supplement: Fig. S2 — Structures of Taniborbactam and Xeruborbactam β-lactamase inhibitors. [file aac.00297-25-s0002.pdf]
