# Supplementary material for: The emerging concern of IMP variants being resistant to the only IMP-type metallo-β-lactamase inhibitor, xeruborbactam
Source: Antimicrob Agents Chemother. 2025 Jun 9;69(7):e00297-25. doi: 10.1128/aac.00297-25 (PMC12217461; doi:10.1128/aac.00297-25)
Supplement: Table S1 — Primers used for site-directed mutagenesis experiments. [file aac.00297-25-s0003.pdf]

MBL mutant	Direction	DNA Sequence (5’-3’)
NDM-1 S262G	For	GAT CGT GAT GGG CCA TTC CGC CC
	Rev	ATG CTG GCC TTG GGG AAC
IMP-14 S47G	For	TAA AGG TTG GGG CGT GGT CAC TAA AC
	Rev	ACT TCT TCA AAC GAA GTA TGA AC
IMP-14 H134N	For	ACA AGC TAA AAA CTC TTT TAA TGG
	Rev	ACC TTA TTG TCT TTT TTA AGA AG
IMP-14 N137S	For	ACA CTC TTT TAG CGG GGT TAG TTA TTC
	Rev	TTA GCT TGT ACC TTA TTG TC
IMP-14 D181Y	For	TGT TAA ACC GTA TGG TCT TGG CTA TTT G
	Rev	AAG CAA CCA CCG AAT AAA ATT TTC
IMP-14 N185Y	For	CGG TCT TGG CAA CTT GGG GGA CG
	Rev	TCC GGT TTA ACA AAG CAA CCA CC

Table S1. Primers used for site-directed mutagenesis experiments.
